# Recurrent Projectile Vomiting as the Sole Presenting Feature of Coeliac Disease With Marsh–Oberhuber Grade 3C Enteropathy in a 13‐Year‐Old Girl: A Case Report

**DOI:** 10.1002/ccr3.73717

**Published:** 2026-10-11

**Authors:** Rahman Syed, Ameer Afzal Khan, Mehtab Alam, Suleman Shah, Mian Gul Rahman, Mohammed Al Meqbaali, Bilal Iqbal, Mohammed Al Sinani

**Affiliations:** ^1^ Swat Medical College Swat Pakistan; ^2^ Saidu Medical College Swat Pakistan; ^3^ Department of Pediatrics Saidu Group of Teaching Hospitals Swat Pakistan; ^4^ Fatima College of Health Sciences Al Ain UAE; ^5^ Imperial College London London UK

**Keywords:** coeliac disease, gluten‐free diet, Marsh–Oberhuber classification, projectile vomiting, villous atrophy

## Abstract

A 13‐year‐old girl presented with 4 months of recurrent projectile vomiting and weight loss without diarrhea. Investigations confirmed hypochloraemic metabolic alkalosis, strongly positive coeliac serology, nutritional deficiency, and biopsy‐proven Marsh–Oberhuber Grade 3C total villous atrophy. A strict gluten‐free diet resulted in clinical resolution and histological recovery to Marsh Grade 1.

## Key Clinical Message

Coeliac disease should be considered in children with chronic vomiting and weight loss, even without diarrhea. Normal hemoglobin and MCV may mask combined nutritional deficiencies. Early coeliac serology and duodenal biopsy are crucial for diagnosis and establishing a baseline for monitoring treatment response.

## 1 Introduction

Coeliac disease is a chronic immune‐mediated enteropathy triggered by dietary gluten in genetically predisposed individuals carrying the HLA‐DQ2 or HLA‐DQ8 haplotype. With an estimated global prevalence of approximately 1.4%, it represents one of the most common chronic gastrointestinal conditions worldwide, though it remains substantially underdiagnosed, particularly in South Asian populations [1]. Classically, the disease presents in childhood with diarrhea, abdominal distension, failure to thrive, and features of malabsorption. However, over recent decades, the clinical phenotype of coeliac disease has shifted considerably, with atypical and extra‐intestinal presentations, including iron deficiency anemia, short stature, elevated liver transaminases, and dermatitis herpetiformis, now outnumbering classic presentations in many series [2, 3]. This evolving epidemiological and clinical picture has been comprehensively summarized in a recent review of pediatric coeliac disease, which highlights the expanding recognition of extra‐intestinal and atypical phenotypes alongside the classical malabsorptive presentation [4].

Recurrent vomiting as the dominant or sole presenting symptom of coeliac disease is uncommon and represents a significant diagnostic challenge, as it directs clinical attention toward upper gastrointestinal motility disorders rather than small intestinal enteropathy [5]. Delayed or missed diagnosis in such atypical cases perpetuates nutritional depletion and disease‐related morbidity. Current ESPGHAN 2020 guidelines emphasize the importance of a low diagnostic threshold in any child with unexplained gastrointestinal symptoms [6]. We report a case of biopsy‐confirmed Marsh–Oberhuber Grade 3C coeliac disease in a 13‐year‐old girl presenting exclusively with chronic projectile vomiting, significant weight loss, and multi‐system nutritional deficiency, highlighting the importance of endoscopic evaluation in atypical presentations.

## 2 Case History/Examination

A 13‐year‐old girl, weighing 27 kg, presented to the Pediatric Department of Saidu Group of Teaching Hospitals (SGTH), Saidu Sharif, with a 4‐month history of recurrent vomiting and progressive weight loss. The vomiting had initially occurred two to three times daily but progressively increased to multiple episodes immediately following every meal. It was projectile in character, yellowish in color, and contained recently ingested food particles, without haematemesis or bile staining. She also reported a dry cough of 4 days' duration prior to admission. There was no history of fever, headache, visual disturbance, photophobia, diarrhea, abdominal distension, jaundice, skin rash, arthralgia, or bone pain.

On physical examination, the patient appeared acutely unwell and markedly wasted, with severe underweight disproportionate to her height. Clinical pallor was evident and early bilateral digital clubbing was noted. There was no peripheral lymphadenopathy, icterus, or pedal oedema. A BCG vaccination scar was present. Vital signs were stable: blood pressure 110/70 mmHg with age‐appropriate pulse and respiratory rate. Oropharyngeal examination revealed hyperaemia of the posterior pharyngeal wall. Chest auscultation demonstrated bilateral equal air entry with no added sounds. Abdominal examination showed a soft, mildly tender abdomen on deep palpation without organomegaly or palpable masses. The remainder of the systemic examination was unremarkable.

## 3 Methods

### 3.1 Differential Diagnosis

Given the presenting complaint of chronic projectile vomiting in a 13‐year‐old without diarrhea, the initial differential diagnosis included: (i) upper gastrointestinal motility disorders, specifically achalasia and esophageal stricture, given the projectile nature and post‐prandial onset of vomiting; (ii) gastro‐esophageal reflux disease with complications; (iii) peptic ulcer disease or gastritis; (iv) psychogenic vomiting; and (v) coeliac disease with atypical presentation, considered in the context of progressive weight loss, pallor, and digital clubbing. The normal esophageal and gastric mucosa on endoscopy excluded motility‐related and structural upper gastrointestinal causes, directing attention to the duodenal findings and confirming coeliac disease as the definitive diagnosis.

### 3.2 Investigations

Table 1 summarizes laboratory investigations. Coeliac serology was assessed by quantitative anti‐tissue transglutaminase IgA (anti‐TTG IgA) enzyme immunoassay and anti‐endomysial antibody (EMA) IgA indirect immunofluorescence, with total serum IgA measured concurrently to exclude selective IgA deficiency.

**TABLE 1:** Summary of laboratory investigations.

Investigation	Result	Reference range	Interpretation
Hematology			
Hemoglobin	15.1 g/dL	12.0–16.0 g/dL	Normal
White cell count	9.31 × 10^3^/μL	4.5–13.5 × 10^3^/μL	Normal
Lymphocytes	50.50%	20%–45%	Elevated
Neutrophils	39.80%	40%–70%	Low‐normal
Platelets	175 × 10^3^/μL	155–450 × 10^3^/μL	Normal
MCV	97.0 fL	80–100 fL	High‐normal
RDW	15.00%	11.5%–14.5%	Elevated
ESR	5 mm/h	0–20 mm/h	Normal
Renal function			
Serum creatinine	0.55 mg/dL	0.2–1.2 mg/dL	Normal
Blood urea	12.1 mg/dL	5–45 mg/dL	Normal
Coeliac serology			
Anti‐TTG IgA	162.4 U/mL	Positive: > 15.0 U/mL	Strongly positive—> 16 × ULN
Anti‐endomysial IgA (EMA)	Positive (1:40)	Negative: < 1:5	Positive
Total serum IgA	235 mg/dL	70–400 mg/dL	Normal—IgA deficiency excluded
Electrolytes			
Serum sodium	136 mEq/L	135–145 mEq/L	Normal
Serum potassium	2.9 mEq/L	3.5–5.1 mEq/L	Hypokalaemia
Serum chloride	91 mEq/L	98–107 mEq/L	Hypochloraemia
Serum bicarbonate	34 mEq/L	22–29 mEq/L	Metabolic alkalosis
Liver function			
ALT	112 U/L	< 35 U/L (female)	Elevated—3.2 × ULN
AST	94 U/L	< 35 U/L (female)	Elevated—2.7 × ULN
Alkaline phosphatase	385 U/L	30–120 U/L	Markedly elevated—3.2 × ULN
GGT	48 U/L	< 38 U/L (female)	Mildly elevated
Total bilirubin	0.7 mg/dL	0.2–1.2 mg/dL	Normal
Nutritional panel			
Serum albumin	2.6 g/dL	3.5–5.2 g/dL	Critically low
Serum iron	24 μg/dL	50–170 μg/dL	Severely deficient
Serum ferritin	6.2 ng/mL	15–150 ng/mL	Severely deficient
TIBC	512 μg/dL	250–450 μg/dL	Elevated—compensatory
Serum folate	1.8 ng/mL	4.6–18.7 ng/mL	Severely deficient
Serum vitamin B12	195 pg/mL	211–911 pg/mL	Below normal
25‐OH vitamin D	11.4 ng/mL	30–100 ng/mL	Deficient
Serum calcium	8.1 mg/dL	8.6–10.2 mg/dL	Low
Serum zinc	48 μg/dL	70–120 μg/dL	Deficient
Viral serology			
HIV	Negative	Negative	Normal
Anti‐HCV	Negative	Negative	Normal
HBsAg	Negative	Negative	Normal

### 3.3 Endoscopy and Histopathology

Upper gastrointestinal endoscopy was performed at the Department of Gastroenterology and Hepatology, SGTH, under topical anesthesia with intravenous domperidone premedication, with biopsies obtained from the second part of the duodenum (D2) for histopathological evaluation. No procedural complications were encountered.

Histopathological analysis was performed at Chughtai Laboratory, Lahore, with formalin‐fixed, paraffin‐embedded sections stained with hematoxylin and eosin and graded according to the Marsh–Oberhuber classification [7]. The specimen consisted of multiple creamish‐white soft tissue shreds measuring collectively 0.4 × 0.3 cm in formalin, processed in a single cassette. The detailed microscopic findings are presented in Table 2.

**TABLE 2:** Histopathological findings.

Parameter	Finding
Specimen	Endoscopic duodenal biopsy—second part of duodenum (D2)
Villous architecture	Loss of normal vertical leaf‐like architecture with severe total to subtotal villous blunting, widening, and extensive surface mucosal flattening
Villous‐to‐crypt ratio	Profoundly inverted—< 1:1 (normal 3:1 to 4:1)
Crypt compartment	Marked vertical elongation, hypercellularity, and widespread crypt hyperplasia
Surface enterocytes	Cuboidal, damaged morphology
Intraepithelial lymphocytes	> 45 per 100 enterocytes (normal < 25)
Lamina propria	Extensively widened with dense chronic inflammatory infiltrate of plasma cells, eosinophils, and small mature lymphocytes
*Giardia lamblia*	Not identified
Acid‐fast bacilli (AFB)	Not identified
Granulomatous inflammation	Absent
Cellular atypia/malignancy	Absent
Marsh–Oberhuber Grade	3C—Advanced Atrophic Lesion
Diagnosis	Severe gluten‐sensitive enteropathy

### 3.4 Treatment

During admission, the patient received intravenous ondansetron (1 mg twice daily), intravenous omeprazole (20 mg once daily), and intravenous Plabolyte M 300 mL eight‐hourly for fluid and electrolyte correction. On discharge, she was continued on oral ondansetron, omeprazole sachets (20 mg once daily), and syrup trimetabol (a non‐hormonal appetite and nutritional stimulant containing metopine, L‐lysine, DL‐carnitine, and vitamins B1, B6, and B12) for nutritional support. A strict lifelong gluten‐free diet was initiated as the definitive therapeutic intervention alongside structured nutritional rehabilitation. Supplementation with iron, folate, vitamin B12, vitamin D, calcium, and zinc was commenced to address the documented multi‐deficiency state.

## 4 Results

### 4.1 Laboratory Findings

Electrolyte studies confirmed a hypochloraemic metabolic alkalosis with hypokalaemia, in keeping with sustained gastric hydrochloric acid and potassium losses from prolonged vomiting (Table 1). Total serum IgA was normal, excluding selective IgA deficiency and validating the IgA‐based coeliac serology, which was strongly positive for both anti‐TTG IgA and anti‐EMA IgA (Table 1). Liver function tests showed transaminase elevation with a disproportionately raised alkaline phosphatase and normal bilirubin (Table 1; see Section 5). The nutritional panel demonstrated a severe multi‐deficiency state affecting albumin, iron indices, folate, vitamin B12, vitamin D, calcium, and zinc (Table 1); notably, hemoglobin and MCV remained within the normal‐to‐high‐normal range despite this deficiency state, with an elevated RDW providing the only routine hematological clue to an underlying dimorphic anemia (see Section 5).

### 4.2 Endoscopic and Histopathological Findings

Upper gastrointestinal endoscopy revealed normal esophageal and gastric mucosa, with no evidence of oesophagitis, erosions, stricture, or dysmotility. The second part of the duodenum showed fissuring of mucosal folds with nodularity; biopsies were obtained without procedural complications. Histopathological examination of the duodenal biopsy specimen confirmed severe villous atrophy with an inverted villous‐to‐crypt ratio, crypt hyperplasia, and marked intraepithelial lymphocytosis, in keeping with Marsh–Oberhuber Grade 3C enteropathy, the most advanced grade of coeliac‐associated mucosal injury (Table 2).

### 4.3 Diagnosis

#### 4.3.1 Outcome and Follow‐Up

The combination of the clinical presentation, strongly positive coeliac serology with normal total serum IgA, and biopsy‐proven Marsh–Oberhuber Grade 3C total villous atrophy established a definitive diagnosis of coeliac disease in full accordance with ESPGHAN 2020 diagnostic criteria [6]. The multi‐system metabolic derangements, hypochloraemic metabolic alkalosis, coeliac‐reactive transaminitis, critical hypoalbuminaemia, dimorphic anemia from combined iron‐folate‐B12 deficiency, fat‐soluble vitamin D deficiency, hypocalcaemia, and zinc depletion collectively reflected the severity and chronicity of the underlying enteropathy.

Following initiation of a strict gluten‐free diet, the patient demonstrated clinical resolution of vomiting, progressive weight gain, and significant symptomatic improvement. Repeat anti‐TTG IgA serology, performed after 8 months, showed a declining titre consistent with sustained dietary compliance and ongoing mucosal healing. Repeat duodenal biopsy after 8 months demonstrated significant histological recovery from Marsh–Oberhuber Grade 3C to Grade 1, with comparative findings presented in Table 3.

**TABLE 3:** Comparative histopathology: baseline versus follow‐up duodenal biopsy.

Histological parameter	Baseline biopsy	Follow‐up biopsy	Change
Marsh–Oberhuber Grade	3C	1	↓ Significant improvement
Villous architecture	Total atrophy—flat mucosa	Restored—near‐normal villous height	↑ Recovered
Villous‐to‐Crypt ratio	< 1:1 (profoundly inverted)	Approaching 2:1–3:1	↑ Improved
Crypt hyperplasia	Marked, widespread	Mild residual	↓ Reduced
Intraepithelial lymphocytes	> 45 per 100 enterocytes	< 25 per 100 enterocytes	↓ Normalized
Surface enterocyte morphology	Cuboidal, damaged	Columnar, recovering	↑ Improving
Lamina propria infiltrate	Dense—plasma cells, eosinophils, lymphocytes	Markedly reduced	↓ Resolved
Giardia/granulomas/malignancy	Absent	Absent	Unchanged

## 5 Discussion

The present case highlights a diagnostically challenging presentation of coeliac disease in which recurrent projectile vomiting, rather than diarrhea, constituted the dominant and sole gastrointestinal complaint over a 4‐month period. Although atypical presentations have become increasingly recognized, vomiting as the primary symptom remains distinctly uncommon and frequently misdirects clinical evaluation toward upper gastrointestinal motility disorders [3, 5]. The prolonged diagnostic delay in this case, during which the patient accumulated significant weight loss and multi‐system nutritional depletion, is consistent with published trends demonstrating that atypical presentations carry substantially longer delays to diagnosis than classical ones [3].

The diagnostic workup fulfilled the ESPGHAN 2020 non‐biopsy diagnostic pathway, given the markedly elevated anti‐TTG IgA with confirmatory EMA positivity and normal total serum IgA (Table 1), as this pathway requires anti‐TTG IgA exceeding 10 times the upper limit of normal with confirmatory EMA positivity [6]. Nonetheless, the additionally performed duodenal biopsy revealed Marsh–Oberhuber Grade 3C total villous atrophy, the most advanced and histologically unambiguous grade of coeliac enteropathy [7]. This degree of mucosal destruction directly accounts for the severity of malabsorption observed across all nutritional parameters and underscores the value of biopsy in establishing disease burden even when serology is already diagnostic.

A particularly instructive finding was the dimorphic hematological pattern: despite severe iron, folate, and B12 deficiency (Table 1), hemoglobin and MCV remained within the normal‐to‐high‐normal range, with an elevated RDW providing the only routine hematological clue. This phenomenon arises when the microcytic tendency of iron deficiency and the macrocytic tendency of folate and B12 deficiency counteract each other, producing a spuriously normal MCV with an elevated RDW as the sole hematological indicator of concurrent combined deficiency [8]. This well‐described but frequently unrecognized pattern in coeliac disease represents a genuine clinical trap: a near‐normal hemoglobin and unremarkable MCV may falsely reassure the clinician, concealing a nutritional crisis of considerable severity. Awareness of this dimorphic pattern in the context of unexplained gastrointestinal symptoms should prompt targeted micronutrient investigation rather than reliance on standard hematological indices alone.

The observed elevation in aminotransferases, together with a disproportionately raised alkaline phosphatase and normal total bilirubin (Table 1), is compatible with coeliac‐associated reactive hepatic involvement, reported in up to 40% of patients with untreated coeliac disease [9]. Rather than reflecting a distinct or diagnostic biochemical signature—no specific pattern of transaminitis is unique to coeliac disease—this reactive transaminitis is thought to arise from increased intestinal permeability with hepatic exposure to dietary antigens and immune complexes. Its resolution following gluten exclusion is well documented and constitutes an additional biochemical endpoint for monitoring treatment response [9].

This trajectory of histological recovery confirmed the reversibility of coeliac enteropathy and the efficacy of strict dietary adherence. In children, mucosal recovery following gluten exclusion typically proceeds more rapidly than in adults, though complete histological normalization may require 12 to 24 months; the residual Grade 1 changes observed here represent an accepted intermediate endpoint reflecting ongoing mucosal healing [10].

## 6 Conclusion

This case underscores that coeliac disease can present exclusively with recurrent projectile vomiting and multi‐system nutritional deficiency in the absence of diarrhea. Clinicians should maintain a low threshold for coeliac serology and duodenal biopsy in any child with unexplained chronic vomiting and weight loss. Early diagnosis, serological confirmation, and strict lifelong gluten‐free dietary adherence are essential to achieve mucosal recovery and prevent progressive nutritional and growth‐related morbidity.

## Author Contributions

**Rahman Syed:** conceptualization, methodology, writing – original draft, writing – review and editing, supervision. **Ameer Afzal Khan:** supervision, writing – review and editing, writing – original draft, methodology. **Mehtab Alam:** methodology, writing – original draft, project administration. **Suleman Shah:** writing – review and editing, writing – original draft. **Mian Gul Rahman:** writing – original draft, methodology. **Mohammed Al Meqbaali:** writing – original draft, writing – review and editing. **Bilal Iqbal:** project administration, validation, writing – original draft. **Mohammed Al Sinani:** writing – original draft, writing – review and editing.

## Funding

The authors have nothing to report.

## Ethics Statement

The authors have nothing to report.

## Consent

Written informed consent for publication was obtained from the patient's parent prior to submission, in accordance with the Clinical Case Reports journal policy.

## Conflicts of Interest

The authors declare no conflicts of interest.

## Data Availability

The data that support the findings of this study are available from the corresponding author upon reasonable request.
